# Continuous Physiologic Markers of Heart Rate Variability Derived From Bedside Electrocardiogram Precede Onset of Acute Respiratory Distress Syndrome: A Physiologic Modeling Study

**DOI:** 10.1097/CCE.0000000000001352

**Published:** 2025-12-10

**Authors:** Curtis E. Marshall, Haoming Shi, Ayman Ali, Victor Moas, Carolyn M. Davis, Jeffrey Wang, Saideep Narendrula, Joao G. De Souza Vale, Jiafeng Song, Hayoung Jeong, Preethi Krishnan, Alasdair Gent, Simon Tallowin, Felipe A. Lisboa, Seth A. Schobel, Eric A. Elster, Timothy G. Buchmann, Christopher J. Dente, Phillip Yang, Rishikesan Kamaleswaran

**Affiliations:** 1 Department of Biomedical Informatics, Emory University School of Medicine, Atlanta, GA.; 2 Emory Critical Care Center, Emory University School of Medicine, Atlanta, GA.; 3 Wallace H. Coulter Department of Biomedical Engineering, Georgia Institute of Technology, Atlanta, GA.; 4 Department of Biomedical Engineering, Duke University, Durham, NC.; 5 Department of Surgery, Duke University School of Medicine, Durham, NC.; 6 Department of Anesthesiology, Duke University School of Medicine, Durham, NC.; 7 Department of Surgery, Emory University School of Medicine, Atlanta, GA.; 8 Department of Medicine, Division of Cardiology, Emory University School of Medicine, Atlanta GA.; 9 School of Biological Sciences, Georgia Institute of Technology, Atlanta, GA.; 10 Surgical Critical Care Initiative (SC2i), Uniformed Services University of the Health Sciences, Bethesda, MD.; 11 Department of Surgery, Uniformed Services University of the Health Sciences and Walter Reed National Military Medical Center, Bethesda, MD.; 12 Academic Department of Military Surgery and Trauma, Royal Centre for Defence Medicine, Birmingham, United Kingdom.; 13 Henry M. Jackson Foundation for the Advancement of Military Medicine, Inc, Bethesda, MD.; 14 Division of Pulmonary, Allergy, Critical Care and Sleep Medicine, Department of Medicine, Emory University School of Medicine, Atlanta, GA.

**Keywords:** acute respiratory distress syndrome, electrocardiogram, machine learning, physiologic monitoring

## Abstract

**OBJECTIVE:**

Acute respiratory distress syndrome (ARDS) is estimated to be prevalent in 10% of ICU patients and results in high mortality rates of up to 45%. The recognition of ARDS can be complex and is often delayed or missed entirely. Recognition of increased ARDS risk among critically ill patients may prompt judicious care management strategies and initiation of preventative therapies known to improve survival.

**DESIGN:**

Retrospective observational cohort study.

**SETTING:**

In-patient tertiary hospital.

**PATIENTS:**

Among 1160 patients (2017–2018), 761 had adequate duration and quality of monitoring waveform data for analysis.

**INTERVENTIONS:**

None.

**MEASUREMENTS AND MAIN RESULTS:**

This is an observational, retrospective, institutional review board-approved study of patients admitted to ICUs at a tertiary hospital system. Physiologic data were captured among critically ill patients who developed ARDS (*n* = 62) and matched controls (*n* = 699) during their hospitalization. Machine learning algorithms were evaluated against statistical features from continuous electrocardiogram (ECG) and sparse clinical data. Waveform-derived cardiorespiratory features, namely measures relating to heart rate variability were found to be robust and reliable features that predicted ARDS up to 2 days before onset. The combined model consisting of waveform features and clinical data with 12-hour prediction horizon achieved an area under the receiver operating characteristic curve and positive predictive value of 0.92 (95% CI, 0.91–0.93) and 0.58 (95% CI, 0.55–0.62), surpassing a model with the clinical data removed (0.86 [95% CI, 0.85–0.88] and 0.49 [95% CI, 0.46–0.52]) and the Lung Injury Prediction Score’s maximum of 0.88 and 0.18.

**CONCLUSIONS:**

Waveform markers can combine with Electronic Medical Records (EMR) data to improve predictability of ARDS before onset. The markers appear to modulate the sparser EMR data. They also provide, in and of themselves, sufficient dynamical information for comparable results to models with EMR data. Further prospective validation is needed to evaluate the robustness of the model and potential clinical utility.

**KEY POINTS**

**Questions:** Can bedside monitors predict acute respiratory distress syndrome (ARDS) onset and provide near real-time monitoring of the risk of progressive respiratory failure among critically ill patients, prompting timely interventions to enhance survival?

**Findings:** Electrocardiogram (ECG)-derived markers reliably predicted ARDS up to two days before onset in this case-control study using predictive machine learning models. The combined model, integrating waveform features and clinical data, achieved an area under the receiver operating characteristic curve of at least 0.92, surpassing the Lung Injury Prediction Score’s maximum of 0.88.

**Meanings:** Integrating ECG-derived markers into predictive models offers a promising method for early ARDS detection, potentially improving patient outcomes through timely intervention.

## BACKGROUND AND SIGNIFICANCE

Acute respiratory distress syndrome (ARDS) is a life-threatening condition in which acute inflammation results in increased pulmonary vascular permeability, pulmonary edema, and severe hypoxemic respiratory failure (1). ARDS is estimated to be prevalent in 10% of all patients admitted to the ICU (2) and is associated with a high mortality rate of up to 45%.

A major barrier to developing therapeutics and management strategies for ARDS is the timely identification of patients who are at risk of developing the condition (3); ARDS diagnosis is often delayed, with 40% of mild cases entirely missed and 21% of severe cases unrecognized (4). This delayed or missed diagnosis can postpone implementation of evidence-informed treatments such as lung protective ventilation and fluid restrictive strategy, contributing to inadvertent harm. These realities lead to a significant interest in its prediction and prevention.

The Lung Injury Prediction Score (LIPS), derived from structured Electronic Medical Records (EMR) and comorbid status, modestly predicted ARDS (area under the receiver operating characteristic curve [AUROC] = 0.70) in a multicenter study (4), but has drawbacks including a high false-positive rate (positive predictive value [PPV] = 0.17) and lacks continuous evaluation during a patient’s stay, typically being calculated with the first six hours of data. Recently, machine learning algorithms using EMR have emerged for ARDS prediction and other critical illnesses like sepsis, but face limitations due to sparse, manually entered data and biases in ordering laboratories and clinical measures (5–9). Analysis of physiologic waveform data from bedside monitors, though continuously monitored in ICU patients, is underutilized in clinical practice. Extracting quantitative markers from routine monitoring to predict ARDS before clinical suspicion could enhance early, unbiased ARDS recognition, improving patient care (10, 11).

Derived from continuous bedside monitoring, “physiomarkers”—electrophysiology and hemodynamic markers—are applied in various areas, such as cardiovascular sufficiency (12), pulmonary fluid status (13), and hypotension (14). Electrocardiogram (ECG) and photoplethysmography-derived markers are particularly useful due to their widespread availability (15). Among various physiomarkers (16–20), the ventilatory ratio (21) stands out for monitoring respiratory instability. Ongoing research explores the potential PPV of autonomic dysfunction markers, particularly low heart rate variability (HRV), for ARDS onset (22–24). Integrating bedside ECG into predictive machine learning models may offer continuous insights into changing respiratory dynamics, aiding critical care management and potentially leading to novel therapeutic avenues, including neuromodulation to ameliorate or halt progression to severe disease.

This study integrates novel physiomarkers from ECG waveform into an explainable machine learning algorithm to predict ARDS onset in critically ill patients 12 hours before onset. The analysis findings, along with a data-fusion model incorporating routine clinical data from the EMR, are reported. The approach in the study is to evaluate waveform data irrespective of the presence of EMR data and test the ability of waveform data to enhance EMR data. Waveform data’s potential to inform about the patient’s condition in the absence of EMR data, and to modulate EMR data. This modulation could compensate for measurement or treatment tendency by hospital or physician, which could have unknown biases. The modulation could also compensate for an individual patient’s unknown biological tendencies (biases) that are not elucidated by sparse EMR data. This waveform data potential is investigated.

## MATERIALS AND METHODS

### Description of Datasets

This is a retrospective observational study using high-fidelity waveform measurements and routinely collected clinical measures from the EMR of patients admitted to Emory University Healthcare ICUs. This study (“Sepsis—AI: Multimodal Study of Sepsis and Related Organ Dysfunction”) was reviewed and approved by the Emory University Institutional Review Board (IRB Number STUDY00000302; approval date: April 29, 2021) and was determined to be non-human subjects’ research, with a waiver of informed consent granted. All procedures were conducted in accordance with the ethical standards of the Emory IRB, consistent with the principles outlined in the Declaration of Helsinki (1975). Accurate characterization of ARDS cases from a retrospective dataset is a major unaddressed challenge (25). To ensure the highest quality of our class labels, expert (including a board-certified intensivist and a surgical resident) physician adjudication using Berlin Criteria was required for positive labeling of ARDS (26). This adjudication included a detailed, independent evaluation of radiographic findings (chest radiograph or CT scan, as explicitly stated) to confirm bilateral infiltrates consistent with ARDS and exclude alternative diagnoses like pleural effusion or pneumonia. In addition, adjudicators meticulously assessed each patient’s medical history, physical examination findings, hemodynamics, clinical laboratory values, echocardiograms, and treatment response to pertinent medications to minimize the influence of confounding factors, particularly focusing on excluding cardiogenic pulmonary edema as a potential cause of respiratory failure. The onset was determined as the earliest timing of at least two consecutive Pao_2_:Fio_2_ ratios less than or equal to 300, and positive end-expiratory pressure greater than 5. Negative labeling of ARDS resulted from failure to meet these criteria or negative adjudication by the experts, as shown in the consort diagram in **Supplemental Figure 1** (https://links.lww.com/CCX/B581).

### Data Processing

Cases included waveform data from 60 hours before onset until 12 hours after, whereas controls had data collected up to 72 hours from a randomly generated window to replicate the uncertainty in ARDS development time and the length of the ARDS patients monitoring. Patients with less than 8 hours of usable waveform data were excluded. Consecutive 8-hour windows of data, with a stride (or incremental step) of 1 h, were used for classification (see **Supplemental Fig. 6**, https://links.lww.com/CCX/B581). Within these windows, statistics were reported for each feature based on measured hourly medians, including mean, median, minimum, maximum, skewness, variance, and kurtosis, whereas discrete events (e.g., administration of a vasopressor) were represented by indicator variables.

Prediction horizons for ARDS were set at 12, 18, 24, 30, and 36 hours before onset, creating a prediction window ending 12 hours post-onset. The windows were used to assign positive labels for case patient data, creating six separate datasets. Overlapping the 8-hour classification window with the prediction window assigned a positive label, whereas the 8-hour windows that did not overlap, including those from control patients, received negative labels. This segmentation follows methods described in a previous study (5) and is documented further in the **Supplemental Document** (https://links.lww.com/CCX/B581).

A full list of the clinical features used in the model is represented in the **Supplemental Table 1** (https://links.lww.com/CCX/B581). Waveform data were obtained from several participating ICUs across the Emory Healthcare System (Atlanta, GA), with ECG sampled at the original sample frequency of 240 Hz using GE Bedside Monitoring (GE Healthcare, Chicago, IL). Non-overlapping 5-minute windows, spaced 10 minutes apart, were selected for feature generation. These features were derived from RR intervals, defined as the time between successive R peaks (representing consecutive heartbeats) in the QRS complex of an electrocardiogram (ECG). The QRS complex is the characteristic waveform reflecting ventricular depolarization during a typical cardiac cycle. Feature extraction was performed using the PhysioNet Cardiovascular Toolbox (27).

Two types of models were compared for each horizon: “waveform(dynamics)-only,” which includes only waveform-derived features, comorbidities, and demographics without traditional clinical EMR dynamics data (i.e., vital signs, laboratories), and “waveform plus EMR” or “combined” uses EMR data alongside waveform-derived features, comorbidities, and demographics. The segmentation of data is logical within the healthcare field, due to comorbidities and demographics nearly always being available during the entirety of patient stay. They are known before a patient’s stay and do not change (static) during the patient’s stay. Thus, they will likely always be used in a predictive model because they will nearly always be available. Waveform data are also likely to be available, especially during the entirety of an ICU visit, but will be dynamic during the patient’s stay. However, the quantity and type of EMR data may be dependent on physician interpretation of the patient’s condition, not to mention physician workload and priorities. Contrasting models with comorbidities (static), demographics (static), and waveform (dynamics) vs. a model that includes dynamic EMR data allows the most realistic potential real-world implementation for evaluation.

There are a total number of 3 demographics, 13 comorbidities, 41 dynamic EMR variables, and 43 dynamic waveform variables. Hourly statistics were calculated on all dynamic variables, except the 11 treatment (discrete events) variables, as described above. Categories of all data are available in Supplemental Tables 1 and **2** for clarification (https://links.lww.com/CCX/B581).

To identify the optimal model for the input data, various learning algorithms were evaluated, including LightGBM (28), Random Forest (RF) (29), and an Artificial Neural Network (ANN) (30). Feature importance and interpretability were characterized using the SHapley Additive exPlanations (SHAP) explainer (31). Model performance was characterized using several benchmarks including AUROC, sensitivity, specificity, and PPV.

## RESULTS

### Patient and Clinical Characteristics

A registry spanning 2017–2018 with 1160 patients and matched waveform data to EMR data was used. Poor waveform quality, largely due to insufficient total monitoring length, removed 399 patients. Among the eligible, 62 were adjudicated with ARDS during their stay, whereas 699 did not meet Berlin Criteria.

Among the final analytic cohort of 761 patients, 62 (8.1%) were adjudicated to have ARDS during their encounter. **Table 1** compares the clinical characteristics of the cohort stratified by the presence of ARDS compared with the absence of the condition. Except for advanced age (older than 80 yr) and a higher percentage of Caucasians in the adjudicated patients, there were no significant differences in age and self-identified race. Compared with non-ARDS patients, ARDS patients had a higher LIPS (mean [interquartile range (IQR), 8.6 [7.6–10.0] vs. 4.4 [3.0–5.0], *p* < 0.001) and lower P/F ratio (127.1 [72.0–166.9] vs. 309.1 [272.5–321.3], *p* < 0.001) consistent with worse oxygenation and lung injury. In-hospital mortality was significantly higher among those with ARDS compared with controls (32.3% vs. 3.4%, *p* < 0.001).

**TABLE 1.** Clinical Characteristics of the Patient Cohort

Category	Total	Acute Respiratory Distress Syndrome	Control	Statistical Significance (*p*)
Patients (*n*)	761	62	699	NA
Males, *n* (%)	408 (53.6%)	30 (48.4%)	378 (54.1%)	> 0.05
Age, mean (IQR)	59.4 (48.0–71.0)	52.6 (42.3–66.0)	59.9 (49.0–72.0)	> 0.05
Age group, *n* (%)				
18–40 yr	124 (16.3%)	14 (22.6%)	110 (15.7%)	> 0.05
41–60 yr	241 (31.7%)	24 (38.7%)	217 (31.0%)	> 0.05
61–80 yr	319 (41.9%)	24 (38.7%)	295 (42.2%)	> 0.05
> 80 yr	77 (10.1%)	0 (0.0%)	77 (11.0%)	> 0.01
Race				
African American	777 (37.4%)	19 (30.6%)	296 (42.3%)	> 0.05
Caucasian	1107 (53.3%)	39 (62.9%)	341 (48.8%)	> 0.01
Asian	53 (2.5%)	0 (0.0%)	19 (2.7%)	> 0.05
Other or unknown	141 (6.8%)	4 (6.5%)	43 (6.2%)	> 0.05
In-hospital deaths, *n* (%)	44 (6.3%)	20 (32.3%)	24 (3.4%)	< 0.001
Hospital length of stay (d), mean (IQR)	12.3 (4.0–14.0)	33.4 (14.0–43.0)	10.4 (4.0–13.0)	< 0.001
ICU length of stay (d), mean (IQR)	6.6 (2.0–7.0)	22.2 (9.3–31.5)	5.0 (2.0–5.0)	< 0.001
Mechanical ventilation, *n* (%)	195 (25.6%)	62 (100.0%)	133 (19.0%)	< 0.001
Vent-free days, mean (IQR)	18.4 (3.0–28.0)	8.0 (0.0–16.8)	23.2 (26.0–28.0)	< 0.001
Lung Injury Prediction Score, mean (IQR)	4.7 (3.0–5.5)	8.6 (7.6–10.0)	4.4 (3.0–5.0)	< 0.001
Tidal volume, mean (IQR)	476.2 (400.0–500.0)	493.3 (400.0–480.0)	462.8 (400.0–500.0)	> 0.05
Tidal volume per predicted body weight, mean (IQR)	7.8 (6.4–8.0)	8.3 (6.1–7.8)	7.4 (6.5–8.2)	> 0.05
Positive end-expiratory pressure, mean (IQR)	8.7 (6.0–10.0)	10.9. (6.0–14.0)	6.4 (6.0–6.0)	< 0.001
Pao_2_/Fio_2_ ratio, mean (IQR)	294.2 (272.5–300.0)	127.1 (72.0–166.9)	309.1 (272.5–321.3)	< 0.001
Spo_2_/Fio_2_ ratio, mean (IQR)	236.4 (221.3–240.0)	122.6 (91.3–155.8)	246.5 (225.0–242.5)	< 0.001
WBC count, mean (IQR)	11.4 (7.5–14.0)	15.5 (8.1–20.8)	11.0 (7.4–13.4)	< 0.001

IQR = interquartile range.

We assessed model performance across 12–36 hours before ARDS onset, generating mean AUROC values and 95% CIs from 30 bootstrapped versions of our three models (**Fig. 1**). As expected, predictive accuracy improved and began to converge as the prediction horizon approached, AUROC peaking 12 hours before ARDS onset (ANN = 0.92; LightGBM = 0.88; RF = 0.86). Further performance metrics are detailed for the ANN models in **Table 2**, demonstrating improved accuracy, sensitivity, and specificity at smaller prediction horizons, albeit with reduced PPV. Notably, the ANN significantly outperformed LightGBM and RF models across the prediction horizon when all features were included, with the most significant difference observed at 36 hours before ARDS onset (ANN AUROC = 0.84; LightGBM AUROC = 0.76; RF AUROC = 0.73).

**TABLE 2.** Results of 12-Hour and 36-Hour Models Using Artificial Neural Network and Random Forest With Lung Injury Prediction Score Comparison

Horizon/Threshold	Dynamic Data Type	Area Under the Receiver Operating Characteristic Curve	Specificity	Sensitivity	Positive Predictive Value
12 hr	ANN waveform	0.86 (0.85, 0.88)	0.97 (0.97, 0.98)	0.75 (0.72, 0.78)	0.49 (0.46, 0.52)
	ANN EMR	0.90 (0.90, 0.91)	0.97 (0.97, 0.98)	0.84 (0.82, 0.86)	0.51 (0.49, 0.54)
	ANN waveform plus EMR	0.92 (0.91, 0.93)	0.98 (0.98, 0.98)	0.86 (0.85, 0.88)	0.58 (0.55, 0.62)
36 hr	ANN waveform	0.80 (0.79, 0.81)	0.98 (0.98, 0.98)	0.62 (0.59, 0.65)	0.61 (0.58, 0.64)
	ANN EMR	0.84 (0.82, 0.85)	0.98 (0.98, 0.98)	0.70 (0.67, 0.73)	0.64 (0.62, 0.67)
	ANN waveform plus EMR	0.83 (0.82, 0.85)	0.98 (0.98, 0.99)	0.68 (0.65, 0.71)	0.70 (0.67, 0.73)
4^a^	LIPS^b^	0.80	0.63	0.97	0.07
5^a^	LIPS^b^	0.85	0.79	0.92	0.11
6^a^	LIPS^b^	0.88	0.89	0.88	0.18

ANN = Artificial Neural Network, EMR = electronic medical records, LIPS = Lung Injury Prediction Score.

a Threshold.

b LIPS was computed using the worst values for features (43).

**Figure 1.**
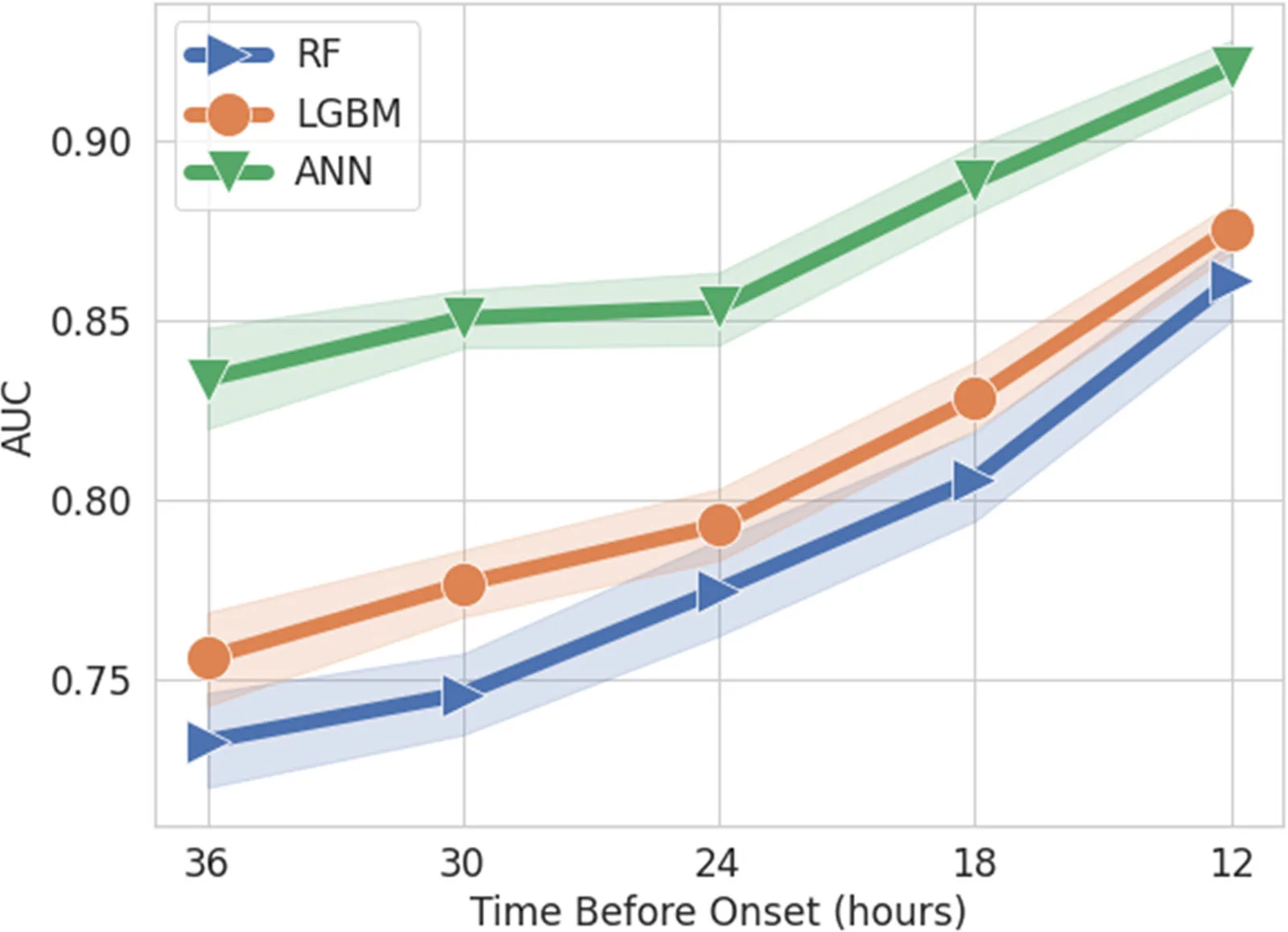
Trajectory of models as prediction horizon approaches onset for the combined features for *Random Forest* (RF), LightGBM (LGBM), and Artificial Neural Network (ANN). AUC = area under the receiver operating characteristic curve.

Next, we compared the predictive accuracy of these combined models to those trained using waveform data alone (**Fig. 2**). The addition of EMR data improves performance consistently across prediction horizon irrespective of the machine learning algorithm used. This improvement in model performance is less pronounced for the RF and ANN models is less pronounced at prediction horizons further from onset.

**Figure 2.**
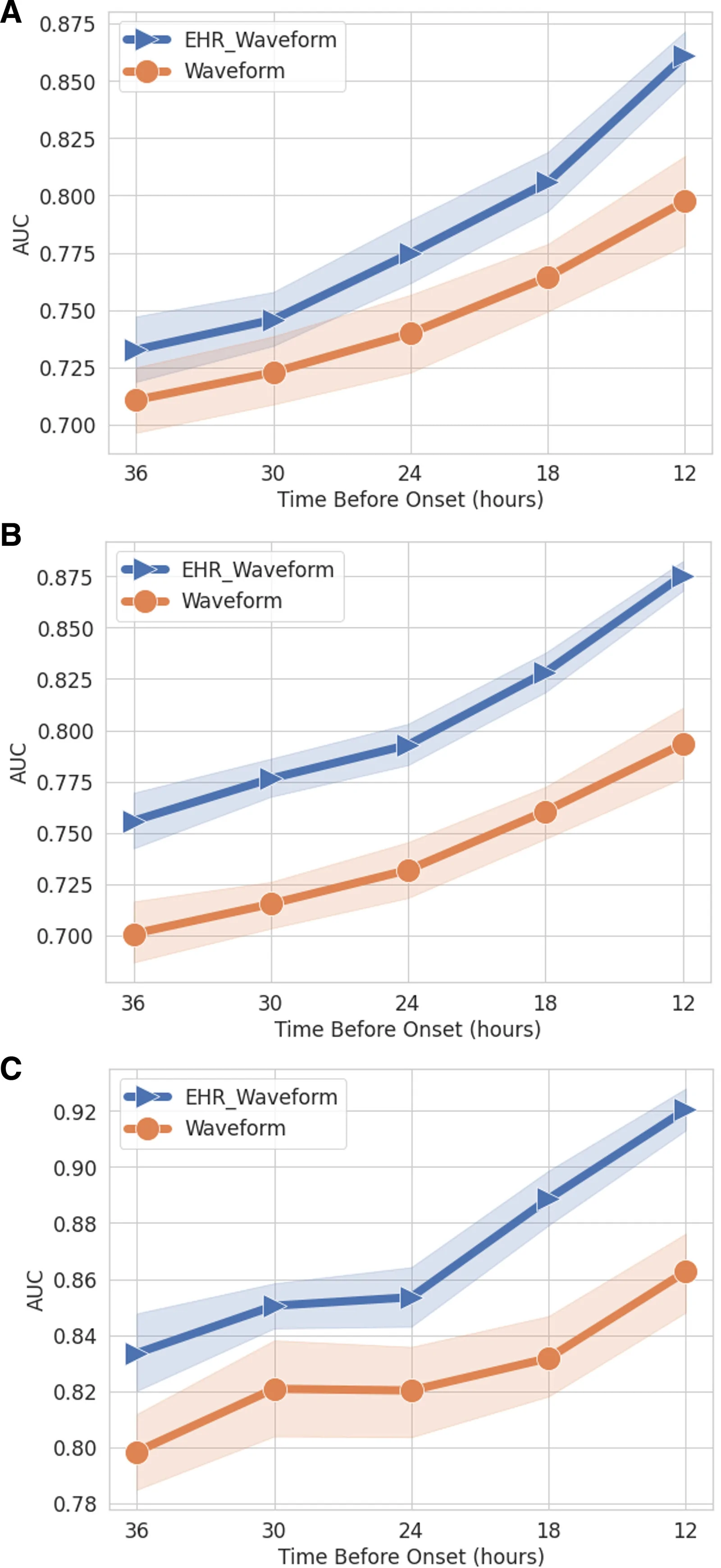
Trajectory of models as prediction horizon approaches onset for the combined features and waveform only for *Random Forest* (**A**), LightGBM (**B**), and Artificial Neural Network (**C**). AUC = area under the receiver operating characteristic curve, EHR = electronic health record.

The best performing model used EMR and waveform features with the ANN algorithm to achieve an AUROC of 0.92, surpassing EMR features with ANN (Table 2, *p* < 0.01). The combined model, also, outperformed the model lacking waveform data in PPV by an even greater percentage (0.58 vs. 0.51). It outperformed LIPS, at multiple thresholds, in AUROC, specificity and PPV. At horizons both close to and further from onset, ANN models significantly outperformed in PPV relative to LIPS (0.58 and 0.70 vs. 0.18). Compared with EMR features alone, the addition of waveform features increased all measures reported for the best-performing model, with the greatest percentage enhancement of PPV at both horizons.

**Figure 3** depicts SHAP values of dynamic variables in the ANN (12 hr before onset) model with combined features (**Fig. 3*A***, defined in Supplemental Document, https://links.lww.com/CCX/B581) and EMR features alone (**Fig. 3*B***). Five of the top 20 combined features are waveform-derived. Kurtosis of pn50 (a measure of the distribution shape for the percentage of consecutive normal-to-normal heartbeat intervals that differ by more than 50 ms), maximum of NN kurtosis (the highest kurtosis value of NN intervals, which indicates the ‘tailedness’ of the heartbeat interval distribution), kurtosis of ApEn (approximate Entropy, a nonlinear measure of signal complexity and regularity), kurtosis of standard entropy of scale 19 (reflecting variability in entropy at a specific scale), and median of pnn50 (the median proportion of NN intervals differing by more than 50 ms relative to the total number of intervals) are waveform features represented in the top 20 dynamic features. In the EMR-only model, there is a much larger presence of mechanical ventilator setting-derived features, and they are higher in importance, as indicated by their order from the top and shown in the magnitude plots in **Supplemental Figure 2** (https://links.lww.com/CCX/B581). The Supplemental Figure 2 (https://links.lww.com/CCX/B581) also shows that the distribution of importance is more balanced in the combined model, whereas in the EMR-only model, the top feature is more dominant. Visual inspection between the two plots shows a distinct decrease in polarity of feature values to model influence when waveform features are included in the model.

**Figure 3.**
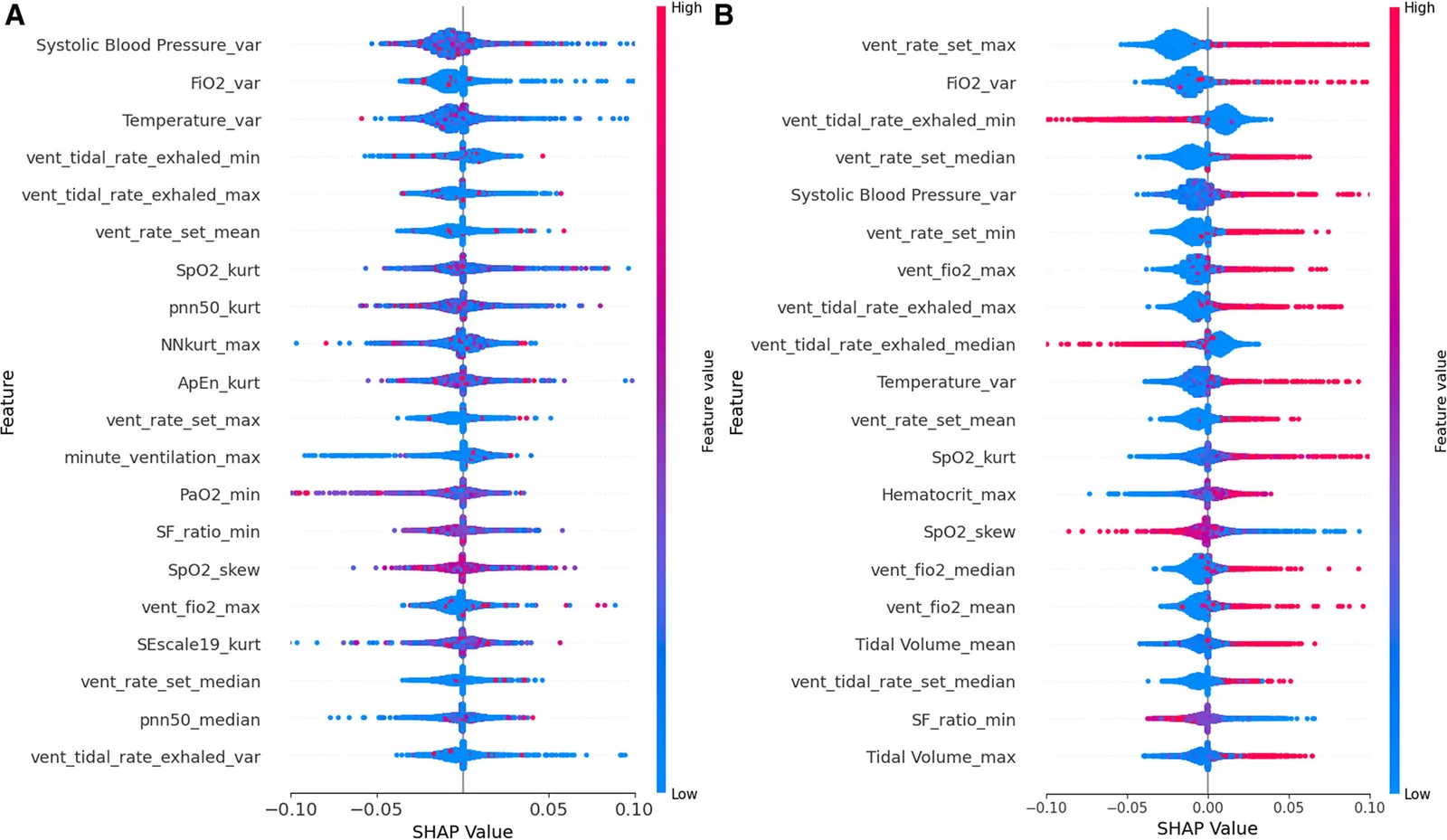
SHapley Additive exPlanations (SHAP) beeswarm of dynamic variables for the combined features (**A**) and electronic medical records only (**B**) for the Artificial Neural Network algorithm at a prediction horizon of 12 h. ApEn_kurt = kurtosis of approximate entropy (ApEn), NNkurt_max = maximum of NN interval kurtosis, pnn50_kurt = kurtosis of pNN50, SEscale19_kurt = kurtosis of standard entropy at scale 19, SF_ratio_min = minimum of the Spo₂/Fio₂ ratio.

## DISCUSSION

This retrospective analysis is the first machine learning application using continuously collected waveform data for the early prediction of ARDS, whose diagnosis is often delayed or missed entirely. Our results show that features solely abstracted from waveform data, without any clinical variables, exhibited robust discriminative performance up to 2 days before patients met the Berlin criteria of ARDS.

The best performing model of this study was achieved by combining waveform data with EMR data in an ANN algorithm with a mean performance of 0.92 at 12 hours before clinical recognition of ARDS. The waveform-only model scored 0.86 AUROC at the 12-hour prediction horizon, indicating that waveform data alone have potential for ARDS prediction. This result could be useful in the pre-clinical and clinical setting in those, rarer, situations in which ARDS develops before, or very early on, in an ICU stay. Perhaps more importantly, it is shown that the combined model scores better in PPV than the EMR-only model at the earliest prediction horizon of 36 h. Furthermore, when applying the algorithms on the patient level to determine patients who met a threshold probability at least once during their stay, the combined model outperformed the EMR-only model. These results suggest that waveform data has a high potential for clinical trial use, when it is important to know those patients who will go on to get ARDS.

The top features of the combined model contained both routine clinical measures, such as dynamic changes in systolic blood pressure, dynamic changes in blood gas measurements, ventilatory support, and dynamic changes in Spo_2_ (Fig. 3). Furthermore, this model consists of several physiologic markers, derived from patient monitoring data. Specifically, pNN50, a hallmark of acute deterioration in several clinical disease profiles, also appeared significant in characterizing the risk of ARDS, as early as 12 hours before clinical recognition. Furthermore, there are other markers, including entropy features and dynamic changes in RR intervals. When comparing the top features for the combined model with the EMR-only model, it is evident that there is a distinct difference in how the feature values affect the model. For example, a high value in the variance of the systolic blood pressure will favor a positive ARDS probability in the EMR-only model, whereas a high value in the combined model may or may not result in a high ARDS probability. It appears that the addition of waveform derived features is modulating the response of EMR features to determine the probability of ARDS in the combined model. These findings suggest a role for physiologic markers as potential enrichment features that not only enable accurate identification of patients with ARDS, but also support a potential unbiased characterization in the presence or the absence of other strongly associated clinical measures such as mechanical ventilator setting-derived features.

Within the scope of predictive models of ARDS, there have been a number of related works. Some have used waveform measures focused only on ventilator-derived waveforms, such as pressure and flow tracing (32, 33). Others have used EMR-based data to predict ARDS without reliance on respiratory physiology and have shown promise (5). Previous work focused on using physiologic signals such as the heart rate and respiratory rate to predict ARDS with an accuracy of 85% 24 hours before ARDS occurrence (34). Similar work based on data fusion focused on using heart rate, respiratory rate, peripheral arterial oxygen saturation, and mean airway blood pressure for early detection (average prediction approximately 39 hr before onset) of ARDS (sensitivity 65%, specificity 100%) (35).

The present model was able to achieve robust discrimination using variables that can be collected almost universally in ICU patients, in contrast to the previous works described above. This waveform data can provide continuous and unbiased indications, and predictive models using such physiologic data can have advantages over the existing models that depend on sparsely collected, biased, and error-prone EMR data. This, in turn, can lead to earlier clinician recognition of ARDS and provide timely clinical decision support for selecting appropriate mechanical ventilation strategies, consideration of adjunctive therapies, and patient selection for ARDS clinical trials.

Despite the recent widespread development of prediction algorithms, there remains a void between reported accuracy and quantifiable clinical impact of clinical decision support systems (CDSS) (32). Recent reviews (36, 37) have concluded that although CDSS can be effective at modifying targeted clinical care processes, there is considerable variation in clinical outcomes and adoption rates. These are critical to overcome alert fatigue and cognitive resistance to established practice that can hinder effective implementation (38, 39). Therefore, a key strength of our approach is the use of routinely collected physiologic markers, coupled with the potential for near real-time prediction. Future work will explore integrating the prediction algorithm into clinical workflows—developing prompts for further assessment and appropriate clinical interventions—while considering potential barriers such as user acceptance and system compatibility. Possible interventions include lung protective mechanical ventilation, fluid restriction, prone positioning, and ventilator synchrony through the use of sedation (40). Another strategy, vagal nerve stimulation, shows promise in modulating inflammatory responses (40). Effective real-time, minute-by-minute monitoring, through waveform data, of the patient condition before, during, and after more established and emerging treatments alike, can be transformative in determining how patients will respond and bridging the response to the underlying biological processes.

There are, however, some notable limitations in this work that need to be overcome to make the bridge possible. The primary concern is the limited number of positive ARDS cases available and the need for validation in an independent dataset. Although expert physician review of ARDS cases enhances model validity and broadens model applicability, it is costly and thus constrains the number of positive cases. A more efficient pipeline for retrospective research would incorporate a comprehensive ARDS identification algorithm, including EMR, radiological notes, and radiological images (25, 41). Furthermore, investigated features reflected HRV through the use of ECG, whereas other routinely collected sensors, such as photoplethysmography, may also provide enriched feature embeddings. Although expert education discriminated against confounding factors (e.g., traumatic injury) that also cause altered HRV, additional data and additional measurement methods would improve future studies of ARDS to prevent false positives and ensure appropriate treatment earlier in, or before, disease progression. A step beyond would include discrimination of the severity of ARDS, which was not attempted in this study due to the limited data size. Machine learning exploration was confined to nonlinear methods and a deep neural network; however, a transformer network that may be able to derive higher-level feature embeddings automatically through feature learning. Exploring these alternatives presents opportunities to refine and expand upon these findings in future research.

## CONCLUSIONS

This study demonstrates the potential of clinical waveform data in predicting ARDS. Results encompassing waveform-only features show strong discriminative performance. Further, features derived from waveform data enhanced prediction at 12 hours before onset, achieving an AUROC of 0.92, a sensitivity of 0.86, and a PPV of 0.58. These findings suggest the possibility of highly predictive markers that only exist within the electrophysiology data, which are often ignored at the bedside. The use of waveform-derived features shows promise both in combination and in the absence of EMR data in earlier recognition of patients with ARDS and may provide less biased and more reliable predictions over exclusively EMR-based predictive models. Such features have the potential to improve bedside clinical care for ARDS and facilitate patient selection for ARDS clinical trials.

## ACKNOWLEDGMENTS

We thank Kim Tierney, Pulakesh Upadhyaya, and Tilendra Choudhary, who helped review the article.

## Supplementary Material
