# Supplementary material for: Continuous Physiologic Markers of Heart Rate Variability Derived From Bedside Electrocardiogram Precede Onset of Acute Respiratory Distress Syndrome: A Physiologic Modeling Study
Source: Crit Care Explor. 2025 Dec 10;7(12):e1352. doi: 10.1097/CCE.0000000000001352 (PMC13652416; doi:10.1097/CCE.0000000000001352)
Supplement: Supplementary file 1 [file cc9-7-e1352-s001.pdf]

Supplemental Figure 1: Consort Diagram of Patient Selection for ARDS Prediction Study

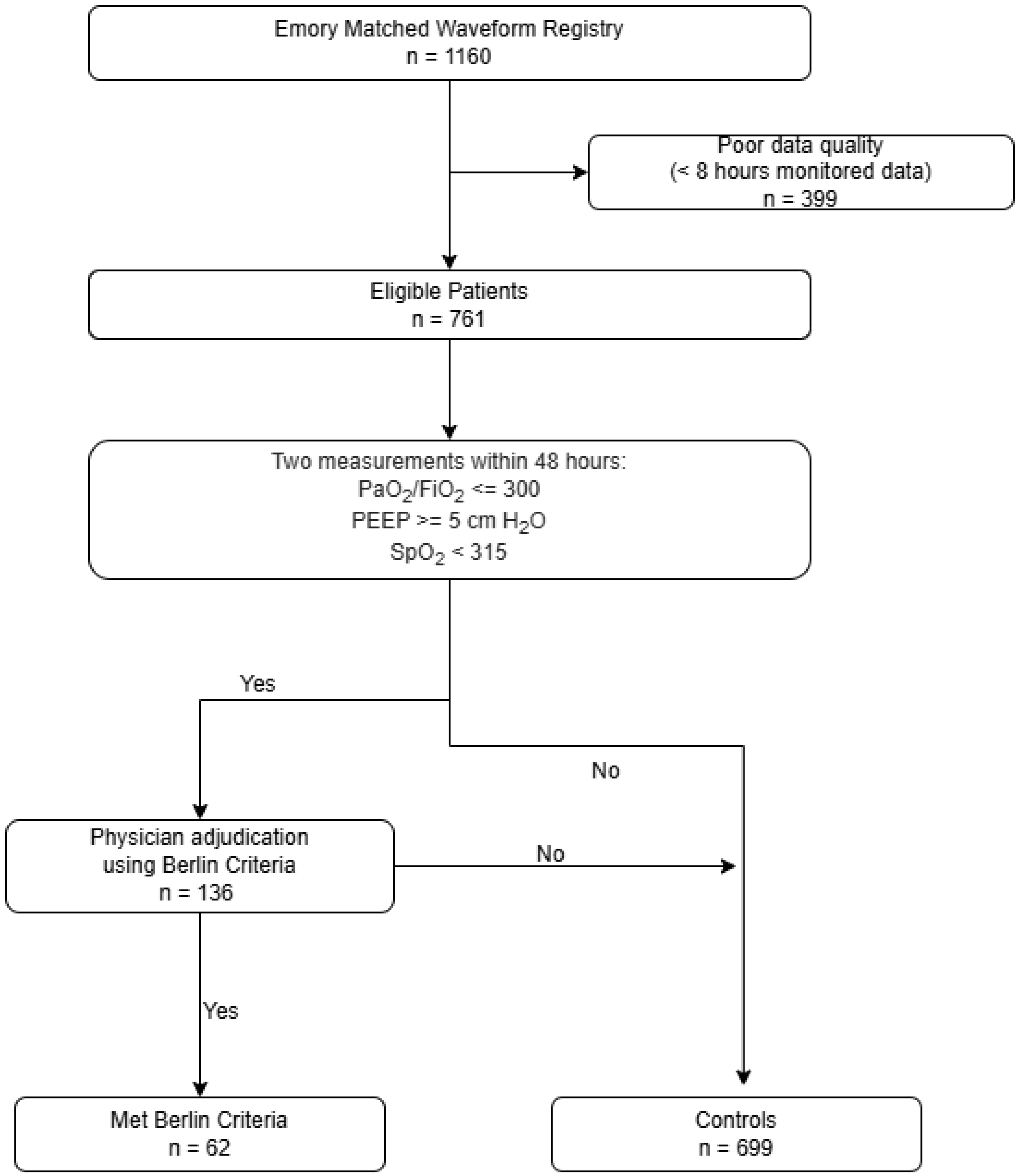

Supplemental figure 1 illustrates the patient selection process for the retrospective observational study. A total of 1,160 ICU patients were initially screened from the Emory University Healthcare database (2017–2018). Patients were excluded based on insufficient duration or quality of high-fidelity waveform monitoring data. The remaining patients underwent expert adjudication using the Berlin Criteria, including radiographic assessment (chest X-ray or CT scan) and clinical review to confirm ARDS diagnosis. Adjudicators excluded patients with alternative causes of respiratory failure, particularly cardiogenic pulmonary edema (CPE). Final classification resulted in 62 ARDS cases and 699 non-ARDS controls, forming the study cohort for machine learning model development and validation.

Artificial Neural Network

The Artificial Neural Network (ANN) described is a sequential model constructed using the TensorFlow Keras library [26]. It consists of three dense (fully connected) layers. The first two hidden layers each have 6 units (neurons) and use the ReLU (Rectified Linear Unit) activation function to introduce non-linearity. The output layer has a single neuron and employs the sigmoid activation function, making the model suitable for binary classification tasks. The model is compiled with the Adam optimizer, using binary cross-entropy as the loss function and accuracy as the evaluation metric. The training process involves a batch size of 5 and runs for 2 epochs.

Supplemental Figure 2: SHAP magnitude plot of dynamic variables for the combined features (A) and EMR only (B) for the Artificial Neural Network algorithm at a prediction horizon of 12 hours.

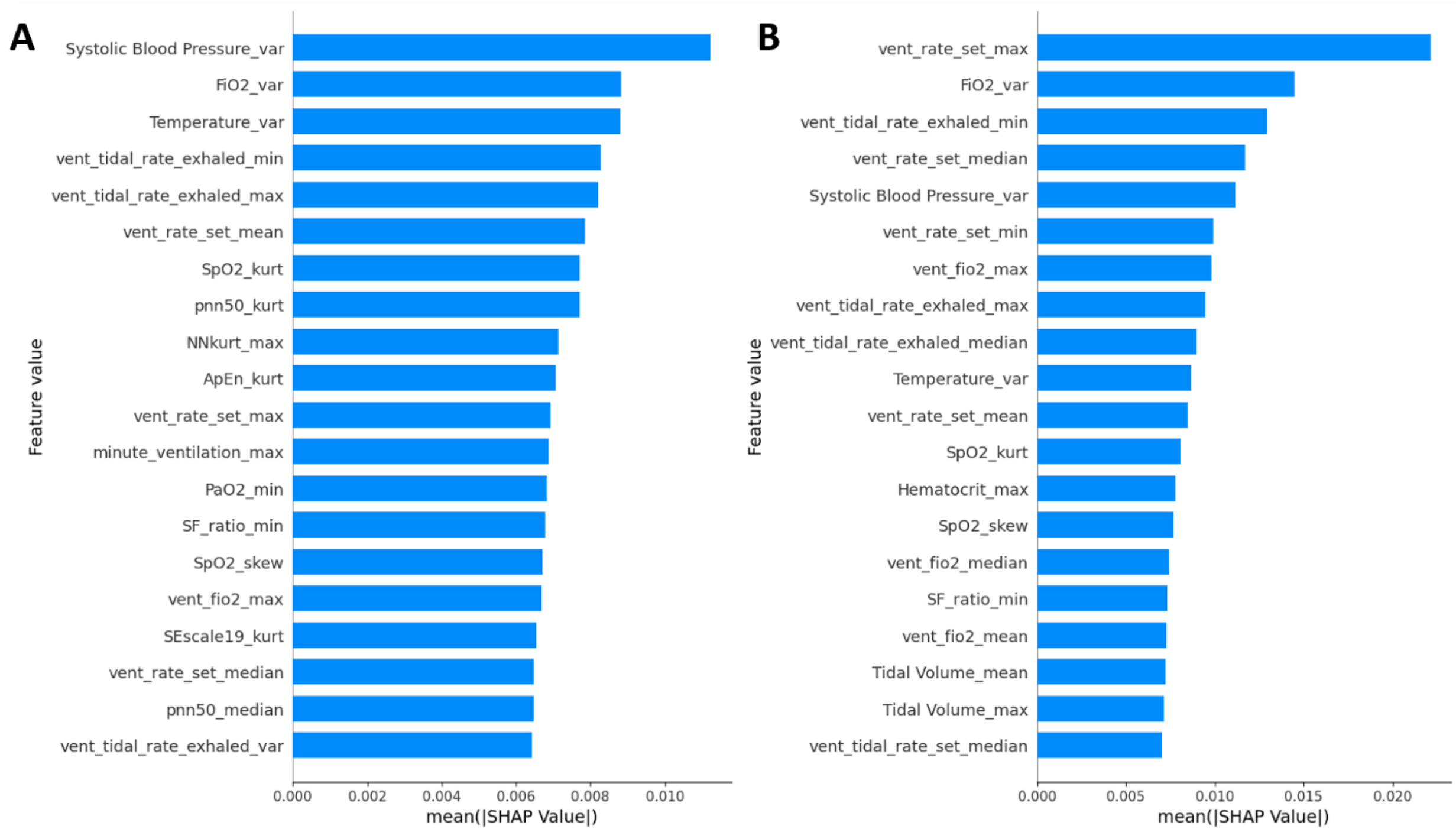

Supplemental figure 2 shows the SHAP magnitude plot for dynamic variables for the combined model and the EMR only model for the artificial neural network at a prediction horizon of 12 hours. This figure compliments figure 3 in the paper. Visual inspection indicates that the EMR only model is much more reliant on the top feature whereas the combined model has a more distributed weighting from the top 20 features.

Supplemental Figure 3: Receiver operating Characteristic and precision recall for Random Forest, LightGBM, and Artificial Neural Network

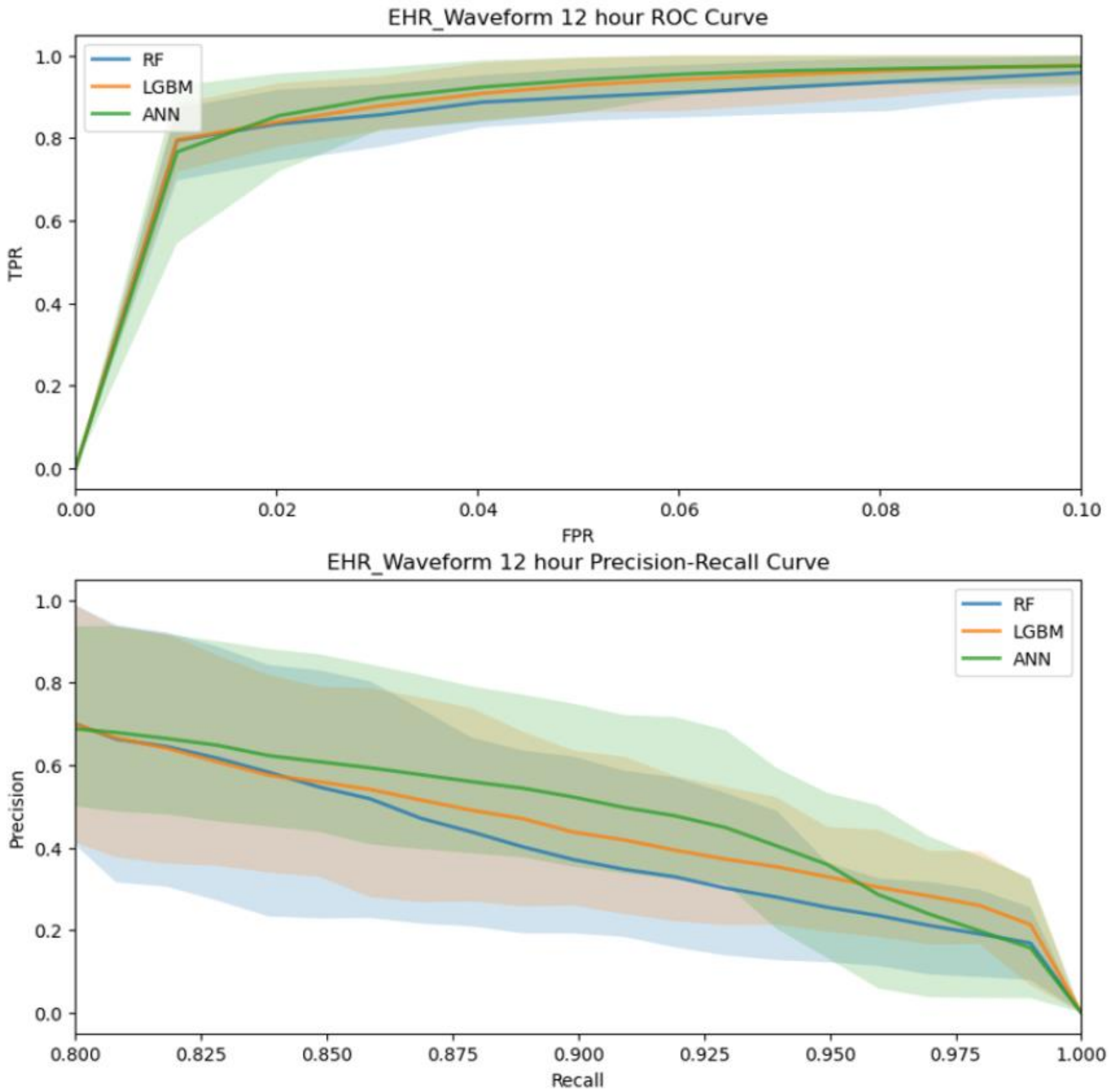

Supplemental figure 3 displays the receiver operating characteristic in the preseason recall curve for the three models in the paper. False positive rates from 0 to 0.1 and recall from 0.8 to 1 are displayed. The ANN model performs best during the majority of values shown.

Supplemental Figure 4: Probability histogram of ARDS (red) and non-ARDS (blue) instances (8 hour windows) in model testing

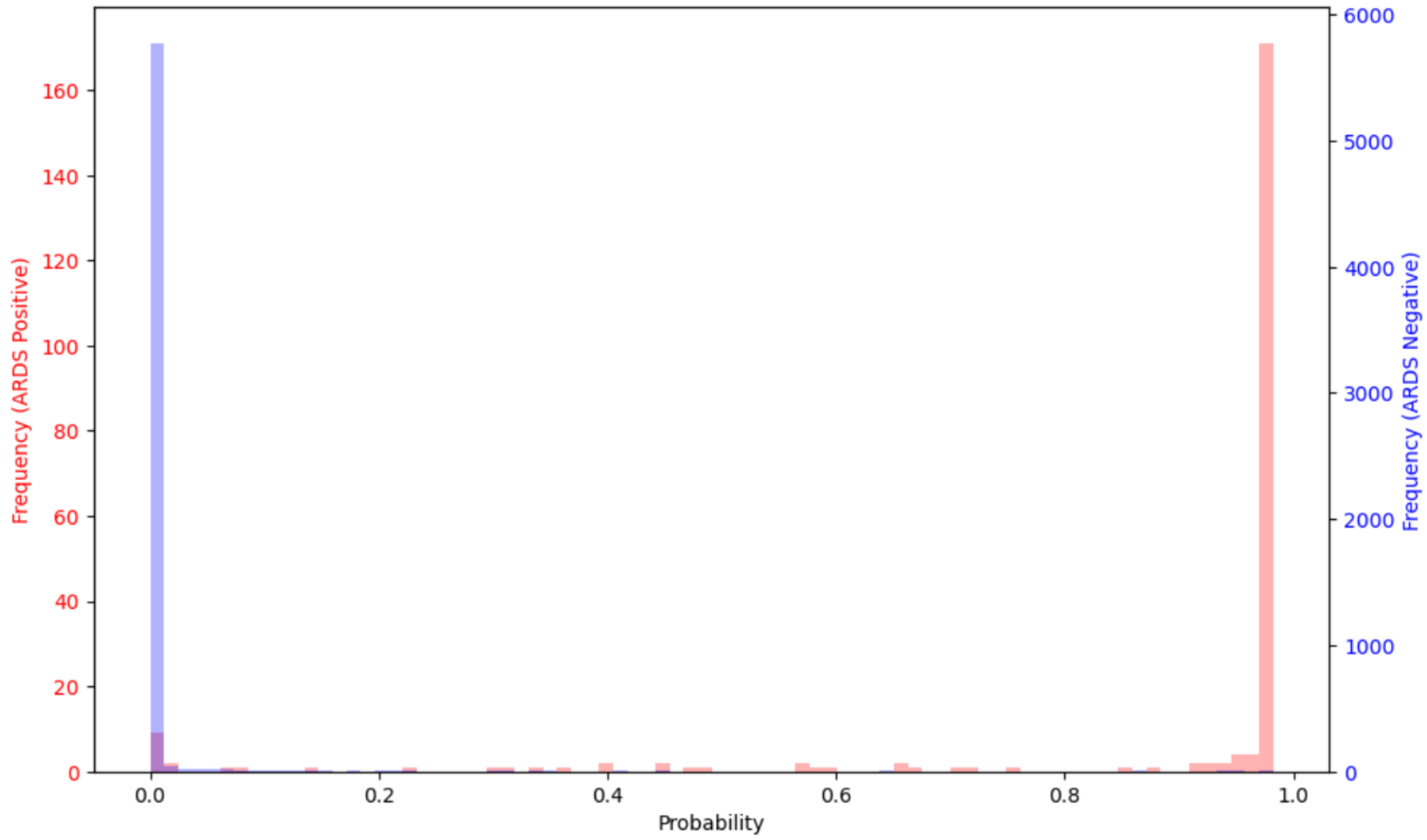

Supplemental Figure 5: Probability histogram of ARDS (red) and non-ARDS (blue) instances (8 hour windows) with mechanical ventilation presence in model testing

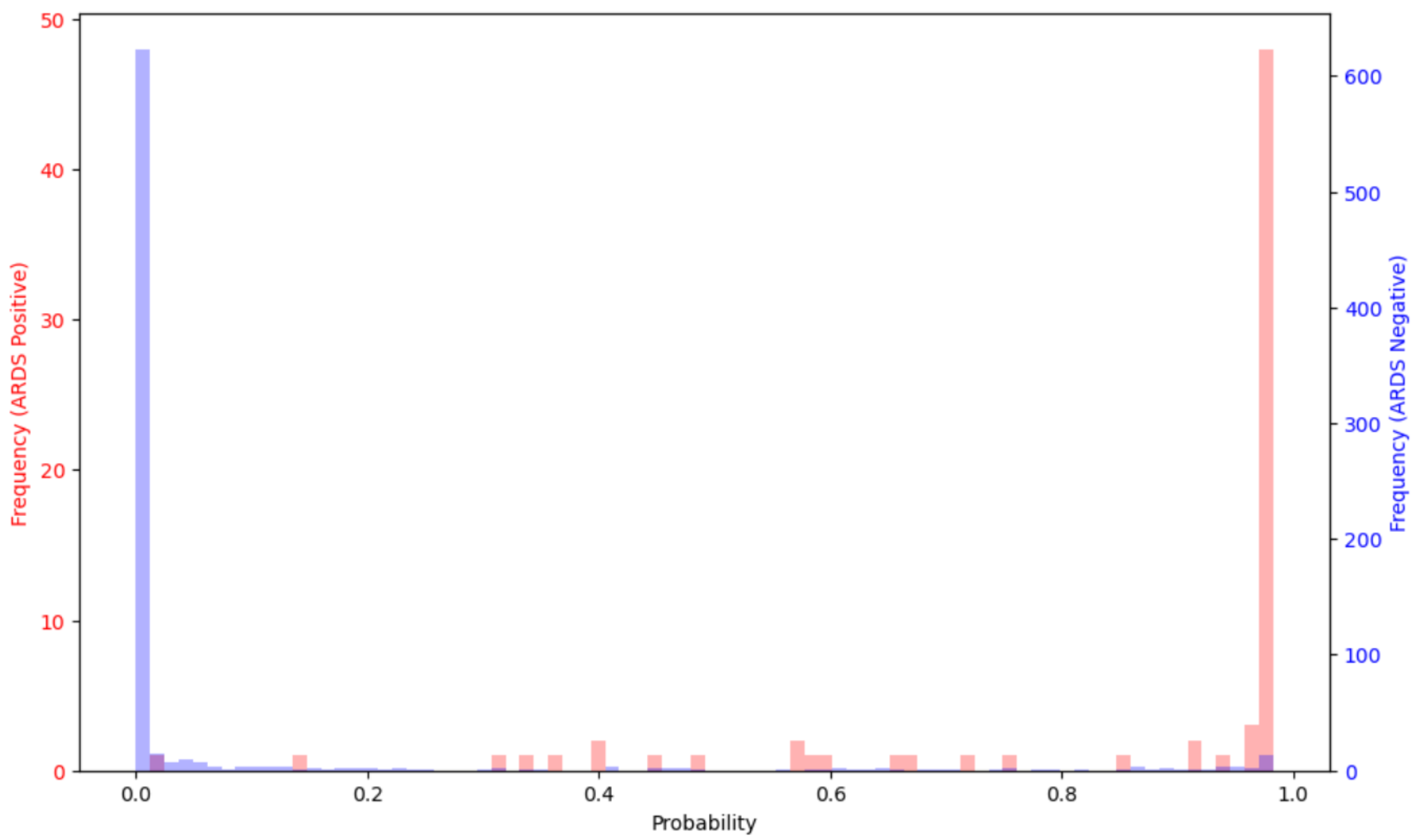

Supplemental figure 4 shows the distribution of probabilities for the test set. The true positives, which correspond to an ARDS physiological, state, or red, and the true negatives are in blue. In supplemental figure 5 is the distribution of probabilities from the same test set but only considering instances with the presence of mechanical ventilation. These distributions show that the model is a skillful classifier, in general, of the ARDS physiological state and is also skillful classifier of the state when only considering instances with the presents of mechanical ventilation.

Data Processing

EMR data included vital signs, lab results, ventilator status and settings, demographics, and existing conditions. A full list of the EMR variables is represented in Supplemental Table 1. For data such as lab results, where there could be a lag time between the collection time and when the results are reported, collection time was used. Preprocessing was performed to remove outliers (most likely due to input errors). Acceptable ranges (with consideration of abnormal condition) for most vitals and lab variables were derived from clinical input. Outing values from these acceptable ranges were removed from the dataset. All data was initially aggregated on an hourly basis. If multiple measurements were taken within an hour for a particular variable, the median value was taken. Statistics were reported for each feature from the 8 hourly values in the window of the case or control. For continuous features, the statistics were mean, median, minimum, maximum, skewness, variance, and kurtosis. This process is depicted in Supplemental Figure 6. For procedures an indicator variable was generated, e.g. admission of a vasopressor, etc.

All waveform derived variables were continuous and were treated in the same fashion as the EMR continuous variables to create features. A total list of variables is available in Supplemental Table 2. Many of the variables can be separated into categories such as time domain, frequency domain, or entropy based.

The ARDS detection model training process begins with data preprocessing, where features are extracted, and numerical values are normalized using MinMaxScaler. Categorical features are processed separately. To prevent data leakage, we employ GroupShuffleSplit, ensuring that the same patient does not appear in both training and testing datasets. The dataset is then divided into 70% training and 30% testing, maintaining strict patient separation. This guarantees that no information from a patient's record in the training set influences the test set, thus ensuring a robust evaluation.

To address class imbalance, SMOTENC generates synthetic samples for the minority class (ARDS physiological state), preventing bias toward the majority class. Multiple machine learning models, including RF, LR, and ANN, are trained with early stopping to prevent overfitting. SHAP values are used for feature importance analysis. For model evaluation, the test dataset is preprocessed separately, with strict patient separation from the training patients set, ensuring that the test samples remain completely independent of the training process.

Supplemental Table 1: EMR Variables

Category	Sub-category	Variable

6 statistics	Vitals	BMI<br>Daily Weight<br>Heart Rate<br>Mean Arterial Pressure<br>Respiratory Rate<br>Systolic Blood Pressure<br>Temperature
	Lab Values	Albumin<br>Bicarbonate<br>Bilirubin<br>Calcium<br>Chloride<br>Creatinine<br>FIO2<br>Glucose<br>Hematocrit<br>Lactate<br>PCO2<br>PaO2<br>Platelet<br>Potassium<br>Sodium<br>White Blood Count
	Ventilator Settings	FIO2<br>GCS<br>PF Ratio<br>Vent Rate<br>Vent Tidal Rate<br>Tidal Volume
	Metric	Glasgow Coma Scale
Maximum value	Concurrent Conditions (comorbidities)	Cardiac Arrhythmia<br>Chronic Pulmonary<br>Congestive Heart Failure<br>Diabetes complicated<br>Diabetes Uncomplicated<br>Hypertension<br>Liver Disease<br>Metastatic Cancer<br>Obesity<br>Renal Failure<br>Rheumatoid Arthritis<br>Valvular Disease
	Treatment	Dobutamine<br>Dopamine<br>Epinephrine<br>Midodrine<br>Milrinone<br>Norepinephrine<br>Number of Concurrent Inotropes<br>Number of Concurrent Vasopressors<br>Phenylephrine<br>Vasopressin<br>Ventilation Duration (Min.)
Demographics	Demographics	Age<br>Gender<br>Race

Supplemental Figure 6: Depiction of Hourly Resampling (A), Accounting for Missing Data (B), Windowing Data for Classification (C), and Statistics Used for Continuous Variables That Make Up Features (D)

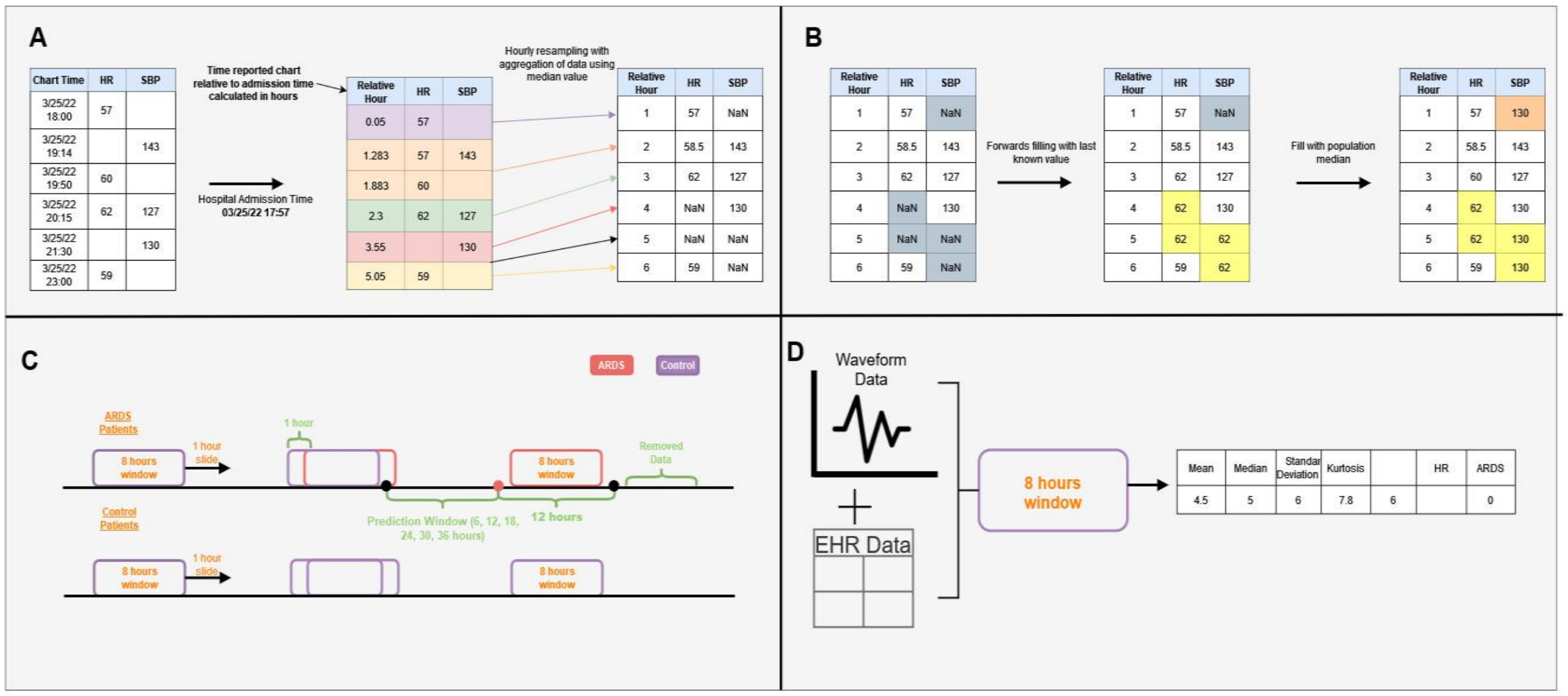

Supplemental Table 2: Waveform Derived Variables

Category	Variable
Time Domain	NNmean
	NNmedian
	NNmode
	NNvariance
	NNskew
	NNkurt
	NNiqr
	SDNN
	pnn50
	RMSSD

Frequency Domain	ulf
	vlf
	lf
	hf
	lfhf
	Ttlpwr
Other	ac
	dc
	SD1
	SD2
	SD1SD2
Entropy	SampEn
	ApEn
	SEscale1
	SEscale2
	SEscale3
	SEscale4
	SEscale5
	SEscale6
	SEscale7

	SEscale8
	SEscale9
	SEscale10
	SEscale11
	SEscale12
	SEscale13
	SEscale14
	SEscale15
	SEscale16
	SEscale17
	SEscale18
	SEscale19
	SEscale20
